# Metastasis-directed therapy for oligometastasis and beyond

**DOI:** 10.1038/s41416-020-01128-5

**Published:** 2020-11-18

**Authors:** Thomas H. Beckham, T. Jonathan Yang, Daniel Gomez, C. Jillian Tsai

**Affiliations:** Department of Radiation Oncology, Memorial Sloan Kettering Cancer Center, Precision Radiation for Oligometastatic and Metastatic Disease (PROMISE) Program, New York, NY USA

**Keywords:** Metastasis, Metastasis

## Abstract

Metastasis-directed therapy (MDT)—local therapy that is intended to eradicate specific metastatic lesions—has hitherto been used with varying degrees of clinical efficacy and acceptance as a meaningful therapy for metastatic disease. Over the past 25 years, however, the momentum for using MDT to manage patients with metastatic solid tumours has increased, driven by several factors. Among these factors is the recognition that patients with limited metastatic burden could potentially derive survival benefits from MDT. Furthermore, although current systemic therapies are increasingly effective, they are infrequently curative. In addition, technological advances have broadened the spectrum of metastatic lesions that can be treated with ablative intent. Here we aim to briefly review the status of evidence for the clinical benefit of MDT based on current data mainly from trials in patients with oligometastatic disease, discuss the myriad of clinical states that might fall under and beyond the definition of oligometastasis, review technological advances in MDT and their applications beyond oligometastasis, and discuss the need for the continued co-evolution of MDT and systemic therapy as we seek to understand which patients with metastatic cancer can achieve durable remission and how to optimally manage those who cannot.

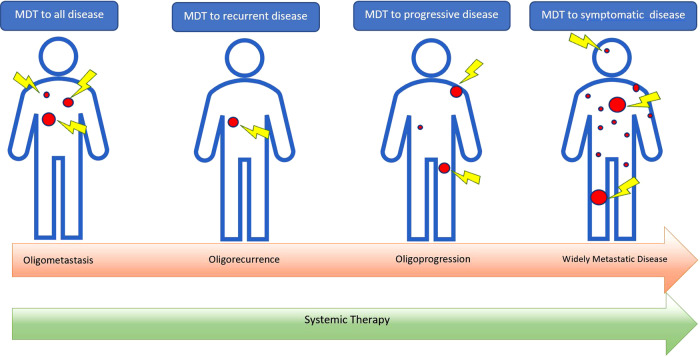

## Background

Cancers that are localised to a primary site are frequently surgically removed or irradiated with curative intent. Unfortunately, many cancers present with or subsequently develop distant metastases, which are generally regarded as incurable. The mainstay therapy for metastatic cancer is typically palliative systemic therapy. Metastasis-directed therapy (MDT)—or local therapy intended to eradicate individual metastatic lesions—extends the principle that local therapy can be curative for localised cancers to patients with metastatic disease. Compelling data exist to support the idea that MDT may meaningfully impact patient survival, and in retrospective experiences a subset of patients have been observed to experience long-term disease-free survival after MDT to limited metastatic foci. Such data have been reported in numerous publications^1–3^ and reviewed elsewhere^4–6^ and so will not be discussed in detail here. Instead, this article will focus on the present understanding of oligometastasis, emerging data on MDT in management of oligometastatic cancer patients, efforts to predict metastatic potential, and appropriate selection of MDT modality.

## Oligometastasis: from proposal to randomised data

The oligometastatic state was described a quarter of a century ago by Hellman and Weichselbaum, who proposed the existence of a state of limited metastasis lying on the spectrum between localised disease and widespread tumour dissemination.^7^

Since this proposal of this state, much effort has been spent debating whether or not such a state exists and whether a definition of oligometastasis can be agreed upon. Recently, a joint effort by the European Society for Radiotherapy and Oncology (ESTRO) and The European Organisation for Research and Treatment of Cancer (EORTC) led to the publication of their categorisation and characterisation of clinical oligometastatic states,^8^ which is driven by five questions about the patient’s clinical history and present disease burden. The result expands upon the umbrella term ‘oligometastasis’ and highlights the existence of nine clinical states that can be considered oligometastasis. Their categorisations are being used to observe the outcomes of patients in a prospective cohort trial, OligoCare (NCT03818503), and will hopefully provide supporting evidence for their characterisations that will inform future therapeutic studies. In April 2020, an ESTRO–ASTRO (American Society of Radiation Oncology) consensus report defined oligometastasis as 1–5 metastatic sites, as well as delivering perspective on other oligometastatic states such as oligoprogression and oligorecurrence, which are discussed more in subsequent sections.^9^ Importantly, both documents highlight that we are in the early stages of defining which patients might benefit from MDT, acknowledging that biological understanding of metastatic potential is needed to truly define oligometastasis, refine patient selection, and inform clinical trial design.

### Clinical studies of MDT

After decades of hypothesis-generating retrospective reports, an important milestone has been achieved with the publication of randomised evidence supporting local therapy for solid metastatic disease. SABR-COMET, an open-label Phase 2 international study, randomised patients with solid tumours and up to five metastases to standard systemic therapy or standard therapy plus stereotactic ablative radiotherapy (SABR) to all metastatic lesions. The study’s primary endpoint of improved overall survival (OS) was met with a median OS of 28 months in the control group and 41 months in the SABR arm.^10^ With longer follow-up, they recently reported a striking 5-year OS benefit for SABR, 42.3% versus 17.7% in the control arm.^11^ A Phase 2 study from the MD Anderson Cancer Center focussed on patients with non-small cell lung cancer (NSCLC) with up to three metastases who did not progress after at least 3 months of first-line systemic therapy, randomising between continued standard of care or local consolidative therapy to all metastatic sites. The study was closed early after the data safety and monitoring board found clear evidence of superiority in the primary endpoint of progression-free survival (PFS) in patients receiving local consolidative therapy. OS, a secondary endpoint, was also improved, with a median of 17 months in the control arm and 41.2 months in the local consolidative therapy group.^12^ Clinical benefits have been shown in other Phase 2 randomised studies, as summarised in Table 1.

**Table 1:** Randomised studies of MDT in oligometastatic cancer.

Publication/name	Population	Intervention	Clinical benefits
Ruers et al.^49^	≤10 unresectable colorectal liver metastases, no extrahepatic disease	Standard systemic therapy versus standard systemic therapy with RFA +/− resection	Hazard ratio for OS^a^: 0.58 (*P* = 0.01)
Gomez et al.^12,50^	*n* = 49^b^, ≤3 NSCLC metastases without progression after 3 months’ systemic therapy	Maintenance chemotherapy versus maintenance chemotherapy after MDT	Median PFS: 14.2 months versus 23.1 months (*P* = 0.022); median OS: 17.0 months versus 41.2 months (*P* = 0.017)
Iyengar et al.^51^	*n* = 29^b^; ≤5 NSCLC metastases with stable disease after induction chemotherapy	Maintenance chemotherapy versus consolidative ablative radiotherapy or SABR followed by maintenance chemotherapy	Median PFS: 3.5 months versus 9.7 months (*P* = 0.01).
Ost et al.^52^	*n* = 62; ≤3 asymptomatic, extracranial prostate metastases	Observation versus MDT (SABR in 25 patients, surgery in 6)	ADT-free survival: 13 months versus 21 months (*P* = 0.11)
Palma et al.^10,11^/SABR-COMET	*n* = 99; ≤5 solid tumour metastases	Standard of care versus SABR + standard of care	Median OS: 28 months versus 41 months (*P* = 0.09) 5-year OS: 42.3% versus 17.7% (*P* = 0.006)
Phillips et al.^14^/ORIOLE Trial	*n* = 54; ≤3 castrate-sensitive prostate metastases	Observation versus SABR	PSA progression at 6 months: 61% versus 19% (*P* = 0.005); median PFS: 5.8 months versus not reached (*P* = 0.002); new lesions at 6 months: 16% versus 63% *P* = 0.006)

*NSCLC* non-small cell lung cancer, *ADT* androgen-deprivation therapy, *PSA* prostate-specific antigen, *RFA* radiofrequency ablation, *SABR* stereotactic ablative radiotherapy, *OS* overall survival, *PFS* progression-free survival.

^a^OS benefit observed on Kaplan–Meier analysis, which was not the endpoint of the study.

^b^Stopped early due to superiority of primary endpoint.

The promising results from early phase studies require confirmation in the Phase 3 setting, and many such trials are underway. NRG Oncology BR002 (NCT02364557), a Phase 2/3 study of MDT for breast cancer in women with ≤4 metastases, has been ongoing since 2015 and met its Phase 2 accrual goal in 2019.^13^ Given the promising data recently published from SABR-COMET,^11^ ORIOLE,^14^ and the MD Anderson study^12^ it is likely that interest in BR002 will be renewed and the rate of accrual will increase. SABR-COMET is being followed up with two Phase 3 studies, both actively recruiting: one for patients with 1–3 metastases (SABR-COMET-3; NCT03862911) and another for patients with 4–10 metastases (SABR-COMET-10; NCT03721341).^15^ The modification of the number of metastases compared with the Phase 2 study is due to the fact that >90% of patients enrolled on SABR-COMET had ≤3 lesions. The expansion of up to 10 metastases in SABR-COMET-10 will allow exploration of the clinical benefits of MDT in patients with more widespread lesions and might give insight into the natural history of metastatic biology both with and without local therapy.

Given compelling evidence from multiple early studies for the successful use of consolidative therapy in patients with oligometastatic NSCLC, multiple randomised trials are recruiting, including the Phase 2/3 NRG LU002 (NCT03137771) and the Phase 3 SARON study (NCT02417662) in the UK. Table 2 includes additional details about these and other randomised studies currently underway that are investigating MDT for oligometastatic and oligoprogressive disease. Built into many of these trials are plans to obtain exploratory biological correlatives, including cell-free DNA, circulating tumour cells, and tissue samples for banking. The outcomes of these and other studies will be crucial in understanding the clinical benefit of MDT, and careful evaluation of biological correlatives will be valuable in increasing our understanding of metastatic biology as well as which patients benefit from MDT (see next section).

**Table 2:** Select ongoing randomised studies of MDT for oligometastatic or oligoprogressive cancer.

Trial ID/name	Population and study design	Accrual target	Intervention	Primary endpoints
NCT02364557/BR002	Phase 2/3; women with breast cancer and ≤4 metastases	*n* = 402	SABR or surgery versus standard of care	PFS (Phase 2) and OS (Phase 3)
NCT03862911/SABR-COMET-3	Phase 3; solid tumour patients with ≤ 3 metastases	*n* = 297	SABR versus standard of care (2:1 randomisation)	OS
NCT03721341/SABR-COMET-10	Phase 3; solid tumour patients with 4–10 metastases	*n* = 159	SABR versus standard of care (2:1 randomisation)	OS
NCT03137771/NRG LU002	Phase 2/3; NSCLC patients with ≤3 extracranial metastases with stable disease after first-line chemotherapy	*n* = 300	Maintenance chemotherapy versus SABR or surgery followed by maintenance chemotherapy	PFS (Phase 2) and OS (Phase 3)
NCT02417662/SARON	Phase 3; NSCLC with ≤ 3 metastases	*n* = 340	Platinum-based chemotherapy versus SABR + platinum-based chemotherapy	OS
NCT02893332/SINDAS	Phase 3; EGFR-mutated NSCLC with ≤5 metastases	*n* = 200	SABR + EGFR-inhibitor versus EGFR-inhibitor	PFS
NCT03808662/CURB- Oligoprogression; PROMISE-004	Phase 2; oligometastatic or polymetastatic NSCLC or breast cancer patients with ≤5 sites of metastatic progression	*n* = 160	Early SABR to sites of progression followed by standard of care versus standard of care	PFS
NCT02756793/STOP	Phase 2; NSCLC with ≤ 5 sites of metastatic progression	*n* = 54	SABR with continuation of current systemic therapy versus physician choice (2:1 randomisation)	PFS
NCT03256981/HALT	Phase 2; NSCLC with response to TKI therapy but progression in ≤3 metastatic sites	*n* = 110	SABR with continuation of TKI versus continued TKI alone	PFS
NCT03599765/EXTEND	Phase 2; solid malignancies with ≤ 5 metastatic sites	*n* = 367	MDT (surgery, radiation, or ablation) then standard of care versus standard of care.	PFS
NCT03410043/NORTHSTAR	Phase 2; stage IIIB/IV EGFR mutant NSCLC not amenable to curative intent therapy	*n* = 143	MDT (surgery and/or radiation) to target lesions after induction osimertinib versus osimertinib alone	PFS
NCT03808337/PROMISE-005	Phase 2; NSCLC or breast cancer patients with ≤ 5 metastases	*n* = 141	SABR versus standard of care	PFS

*EGFR* epidermal growth factor receptor, *TKI* tyrosine kinase inhibitor, *NSCLC* non-small cell lung cancer, *SABR* stereotactic ablative radiotherapy, *OS* overall survival, *PFS* progression-free survival.

## Biological characterisation of metastatic potential

### Predicting metastatic potential

In an era of increasing understanding of the molecular underpinnings of malignancy, the identification of ‘drivers’ of metastasis has been elusive. This is attributable to the biologically complex set of cellular behaviours required for metastasis,^16^ the poorly understood relationship between the tumour-intrinsic features that facilitate this process and the tumour microenvironment that supports it,^17,18^ and the limited capacity of mutation-based analysis to explain and predict complex phenotypes. A remarkable effort by Priestley et al. underscores the limitations of genomic analysis in predicting the metastatic behaviour of solid tumours.^19^ The researchers analysed whole genomes of >2500 paired primary and metastatic tumour samples and uncovered no significant underlying differences between the genomes of primary and metastatic tumours; nor were any mutational signatures for metastasis found. On the other hand, many investigators have searched for metastasis genes in more focussed investigations of specific diseases, with some early success (reviewed in ref. ^20^), and it is clear from sequencing-based studies that alterations in specific pathways might be associated with patterns of metastasis and the overall tempo of metastatic disease.^21^ However, although genetic alterations associated with metastasis have been found in hypothesis-generating studies, it is widely recognised that the processes underlying metastasis—and thus metastatic potential—are too complex to be predicted based on genomic alterations alone.

Despite the inherent challenges, progress has been made in biologically defining metastatic potential. Twenty-five years after proposing the term oligometastasis, the Weichselbaum group made perhaps the greatest contribution to date in improving upon predictive clinical features to stratify the outcomes of patients with colorectal cancer after MDT. Using metastatic tumour tissue from patients with colorectal cancer undergoing hepatic metastasectomy for limited liver metastases, the group used a technique known as similarity network fusion analysis^22^ to cluster patients based on similar expression profiles of both messenger RNA (mRNA) and microRNA (miRNA) in their resected metastatic tissue.^23^ The resulting three molecular signature profiles were found to significantly improve upon a well-validated clinical risk score used for selecting patients for MDT in colorectal cancer. Such a strategy serves as a proof-of-concept that we can predict biological behaviour using multiplatform molecular analysis. It also highlights the challenges ahead. DNA sequencing, mRNA expression, and miRNA expression from the primary tumours, as opposed to the metastases, could not improve upon clinical features, suggesting that metastatic potential might be challenging to predict using primary tumour tissue even with integrated molecular analysis. Moreover, it appears that complex and costly analysis is likely to be required to derive molecular descriptions of metastatic potential. Another impressive contribution sequencing the exomes of primary tumours and brain metastases revealed that most actionable mutations in brain metastases were not evident in the primary tumour sample.^24^ The identification of actionable mutations in brain metastases is promising, particularly as targeted therapies are showing more efficacy in this setting;^25^ however, the inability to infer such mutations from primary tumour tissue emphasises the challenges of understanding metastatic potential from primary tumour tissue and thus limits efforts to personalise therapy.

### Progress with biological correlatives

As we seek a deeper understanding of the molecular underpinnings of metastasis and biological features that could help select patients for MDT, it is also critical to maximise information obtained from completed and ongoing clinical trials. For instance, Tang et al. evaluated circulating tumour DNA (ctDNA), cytokines, and the T cell repertoire from biospecimens collected during the Phase 2 study of NSCLC patients receiving MDT after induction chemotherapy and found that MDT was associated with a reduction in ctDNA and the expansion of a subset of T cell clones. Interestingly, in a small subset of patients from whom serial ctDNA samples were taken, a rise in ctDNA mutation burden significantly predated clinical progression of the disease.^26^ Biological correlatives are built into the majority of ongoing studies in Table 2. Hypotheses generated from such analyses will provide valuable insight into the systemic response to MDT and how clinicians evaluate the success of MDT and monitor disease recurrence.

## Above and beyond oligometastasis

### MDT in disease control

The development of trials and identification of patients who would benefit from MDT are currently limited by a simplistic definition of oligometastasis and minimal understanding of what drives metastatic biological potential. The most important among these, and inherent to the term itself (from the Greek ‘oligos’ meaning ‘scant, few’), is the number of metastases. Although it is pragmatic to consider patients with a limited number of metastases for MDT, the advancement of radiotherapy technology has the potential to broaden MDT indications to those with numerous metastases. In such cases, it might become necessary to reassess the goal of MDT and consider the benefits beyond eradicating specific metastases. While the intention of ablative therapies is to kill or remove a metastasis completely, systemic treatments for metastatic cancer patients usually have a more modest, albeit still valuable, goal of disease control. Palma et al. pose an intriguing question of whether administering MDT in the form of SABR to more widespread metastases might be a meaningful disease control strategy.^27^ Even if ablative doses to all lesions are not achieved, which is a likely prospect when considering radiation for numerous lesions in diverse locations, lower doses of radiation are usually feasible and provide meaningful local control. SABR-COMET-10 is pushing the boundaries of this concept and should provide insight into whether the limits that determine which patients we consider for MDT should be pushed even further.

Definitions of the array of clinical states for which the prefix ‘oligo-‘ might be applicable are comprehensively considered in the ESTRO–EORTC and ESTRO–ASTRO joint reviews,^8,9^ but these definitions expand upon concepts that are already gaining clinical momentum. Oligoprogressive disease, wherein patients with polymetastatic disease experience progression in limited foci, is a compelling situation in which to consider MDT. In the current era of molecularly targeted systemic therapy, increasingly effective and tolerable options for disease control are available. In a widely metastatic patient on a tolerable regimen controlling the majority of their disease, MDT might be an attractive alternative to switching systemic therapies, particularly given the general paradigm that subsequent lines of therapy are less likely to be effective. Additionally, with few exceptions, molecularly targeted therapies are effective at controlling, but not eradicating, disease and are characterised by the emergence of resistance.^28^ In patients with controlled but persistent metastases on targeted therapies, consolidation with MDT could offer an opportunity for the elimination of bulky reservoirs of disease, which might, in turn, limit the emergence of resistant clones or offer an opportunity for a treatment holiday. Our centre is actively recruiting to a randomised trial for patients with oligoprogressive breast and lung cancer (PROMISE-004, Table 2). Other studies testing this approach for NSCLC (NCT04216121), renal cell carcinoma (NCT03696277, NCT04299646), and prostate cancer (NCT04070209, NCT04222634) are underway.

### SABR and immunotherapy

Checkpoint blockade therapy has been another paradigm-changing approach in the treatment of multiple malignancies at various stages, particularly in metastases. However, response rates to immunotherapy in most solid tumours remain disappointing and many patients do not have durable responses.^29^ Incorporation of MDT as a consolidative therapy in patients with partial responses to immunotherapy might become a useful therapeutic strategy and is being tested prospectively at our institution (NCT03693014). In response to reports of abscopal responses achieved with the addition of SABR to immunotherapy,^30,31^ potential synergy between local therapies such as cryoablation or SABR in promoting systemic antitumour responses remains of great interest.^32,33^ Moreover, combining stereotactic radiosurgery with immunotherapy for melanoma brain metastases has been observed to be safe and to potentially promote regression of the irradiated lesion itself.^34,35^ The potential synergy between radiotherapy and immunotherapy is receiving significant attention in the preclinical setting, but randomised data have not yet proved whether this approach provides meaningful benefit to patient outcomes.^36^

### MDT and symptom management

Although MDT therapy holds substantial promise in improving the survival of patients, it is also important to consider the important roles MDT plays in symptom management. Radiotherapy has numerous roles in managing pain, bleeding, and mechanical obstruction from metastases. These well-established indications are outside of the scope of this review, but developing indications for MDT in preventing and managing adverse events is worth mentioning. One such indication is the prevention of skeletal-related events that can result from metastatic progression, such as spinal cord compression or long bone fracture. Skeletal-related events result in the use of significant healthcare resources,^37^ and the prevention of skeletal-related events has been shown to reduce healthcare costs.^38^ Our centre is carrying out a randomised study comparing standard of care versus palliative radiotherapy for asymptomatic but high-risk skeletal metastases, with a primary endpoint of reducing the rate of skeletal-related events (NCT03523351). Although most of this review focusses on the roles of MDT in prolonging OS or PFS, quality of life and healthcare-cost-related endpoints are important parameters to consider as trials intended to prevent adverse events from metastases are developed.

## Local therapy for metastatic disease

MDT intended to remove or ablate individual metastases has become increasingly feasible. The earliest evidence of MDT comes from a surgical series of pulmonary^2^ and hepatic^1^ metastasectomies. The feasibility of applying existing surgical techniques to metastatic disease laid the groundwork for demonstrating that aggressive MDT might confer a worthwhile benefit. In a number of settings, surgery remains the best option for MDT. However, given the complexity of metastatic behaviour and the number of organ systems that can be involved, there has been an effort to develop minimally or non-invasive methods for delivering definitive MDT.

### Radiofrequency ablation (RFA)

A number of interventional techniques have gained acceptance as ablative modalities for metastatic tumours. Thanks to advances in image guidance, percutaneous approaches to targeting liver metastases with thermal or RFA have become increasingly popular. The largest published experience of RFA reported particular success for tumours <3 cm in diameter and in those for which a 10-mm margin of ablation could be achieved.^39^ In lesions with a 10-mm ablation margin, long-term local control was >90%. Likewise, pulmonary metastases are accessible percutaneously for interventional ablative procedures. In a study of >1000 metastases to the lung treated with RFA, local control at 4 years was 89% and was better in patients with smaller tumours.^40^ Interventional approaches are minimally invasive, but they do still facilitate tissue sampling prior to ablation, which is an advantage in scenarios where diagnosis is in question or additional tissue might be desired for molecular analysis.

### Stereotactic ablative radiotherapy

External beam radiation therapy has benefitted from cumulative advances in imaging, simulation, and treatment technology to lead to the widespread adoption of SABR throughout the field. Although definitions of SABR vary, the essential implication of the term is delivery of high doses of highly conformal radiation over 1–5 treatments. SABR is non-invasive and, with few exceptions, can be adapted to ablate a large majority of lesions. Success with SABR has been clearly demonstrated in diverse clinical settings. In a pair of Phase 1/2 studies of SABR using 60 Gy in three fractions, 2-year local control for patients with lung metastases was 96%^41^ and for patients with liver metastases it was 92%.^42^ In studies of spinal metastases, rates of local control are typically >80%;^43,44^ however, tumours irradiated with higher doses can demonstrate much better local control. In a large retrospective experience analysing dosimetric parameters of 811 spinal tumours treated with single-fraction SABR, those treated with ‘high dose’ SABR with a median GTV D95 (dose to 95% of the gross tumour volume) of 23.56 Gy were found to have 4-year local control of 98%, whereas lesions in the ‘low dose’ group, defined as GTV D95 17.09 cGy, had approximately 80% 4-year local control.^45^ The same study also showed that SABR effectively overcomes issues of inherent radioresistance of some histologies, with classically radioresistant metastases such as colorectal, renal cell carcinoma, and sarcoma showing similar local control to patients with classically radiosensitive histologies like breast and prostate. Additional experiences in SABR for oligometastases are well reviewed^46^ and outside the scope of this manuscript. In general, however, SABR offers a non-invasive, adaptable, and well-tolerated option for the management of metastases in a wide array of organs. Safe dose escalation to truly ablative levels has been achieved in most organ sites with improved simulation, image guidance and motion management, and the use of advanced radiation treatment delivery techniques.

### MDT and adverse events

Although the feasibility, tolerability, and options for MDT have expanded, no MDT without some risk exists. The rates of high-grade adverse events from SABR studies are typically low, but SABR-COMET recorded three fatal toxicities related to the receipt of ablative radiation. In the previously discussed series of RFA for lung metastases, 58% of patients required a chest tube for pneumothorax management, and 25% were hospitalised for ≥4 days after the procedure, with 2 deaths in the postoperative period.^40,47^ Shady et al. reported a 7% rate of ‘major’ complications of RFA for liver metastases.^39^ Surgical metastasectomies carry site-specific perioperative risks. In the classic report of hepatic metastasectomy for colorectal metastases in 1001 patients, Fong et al. reported a median hospital stay of 11 days and a 2.8% rate of perioperative mortality,^1^ whereas Pastorino et al. recorded a 1.3% rate of perioperative death in 5206 cases of pulmonary metastasectomy.^2^ These risks highlight the need for a better understanding of patients who are most likely to benefit from MDT so that patients and providers alike can understand the risks and benefits of therapy. Moreover, selection of the appropriate modality of MDT and its skilful delivery are paramount as we seek to maximise the therapeutic ratio of MDT. Dedicated centres are increasingly recognising the value of the multidisciplinary management of patients with metastases in specialised sites such as the central nervous system. Memorial Sloan Kettering Cancer Center has developed a metastatic disease programme within The Department of Radiation Oncology—The Precision Radiation for Oligometastatic and Metastatic Disease (PROMISE) Program—that is intended to ensure specialist-level consideration of the use of radiation in metastatic patients and to support the development of clinical trials and supportive research into the use of MDT.^48^

## Conclusions

Modern MDT encompasses numerous versatile modalities that have the potential to positively affect the outcomes of patients with all manner of metastatic disease burden, with compelling data from small studies suggesting overall survival benefits in patients with oligometastasis treated with MDT. Building on this early success, Phase 2 and 3 studies are underway to more definitively test MDT in oligometastasis as well as in other states such as oligoprogressive disease and more widespread cancer dissemination. These studies will help us understand the clinical scenarios in which MDT meaningfully improves patient outcomes. Continued investigation into the molecular mechanisms of metastasis might help us to refine our definitions of oligometastasis, by going beyond clinical features alone and incorporating an understanding of biologic metastatic potential. Patients with metastatic cancer now have more effective systemic options for managing their disease, and the tailored incorporation of MDT for consolidation and oligoprogression holds promise for improving the length and quality of life for many individuals with advanced cancer.

## Data Availability

Not applicable.
